# Kinesin-5 inhibition improves neural regeneration in experimental autoimmune neuritis

**DOI:** 10.1186/s12974-023-02822-w

**Published:** 2023-06-09

**Authors:** Felix Kohle, Robin Ackfeld, Franziska Hommen, Ines Klein, Martin K. R. Svačina, Christian Schneider, Gereon R. Fink, Mohammed Barham, David Vilchez, Helmar C. Lehmann

**Affiliations:** 1 Department of Neurology, Faculty of Medicine, University of Cologne and University Hospital Cologne, Cologne, Germany; 2 Cologne Excellence Cluster on Cellular Stress Responses in Aging-Associated Diseases (CECAD), University of Cologne, Cologne, Germany; 3 Institute of Neuroscience and Medicine (INM-3), Cognitive Neuroscience, Research Center Juelich, Juelich, Germany; 4 Department II of Anatomy, Faculty of Medicine, University of Cologne and University Hospital of Cologne, Cologne, Germany; 5 Faculty of Medicine, Center for Molecular Medicine Cologne (CMMC), University Hospital of Cologne, Cologne, Germany; 6 Department of Neurology, Hospital Leverkusen, Leverkusen, Germany

**Keywords:** Experimental autoimmune neuritis, Guillain–Barré syndrome, Autoimmune neuropathy, Eg5, Monastrol, Neuroregeneration

## Abstract

**Background:**

Autoimmune neuropathies can result in long-term disability and incomplete recovery, despite adequate first-line therapy. Kinesin-5 inhibition was shown to accelerate neurite outgrowth in different preclinical studies. Here, we evaluated the potential neuro-regenerative effects of the small molecule kinesin-5 inhibitor monastrol in a rodent model of acute autoimmune neuropathies, experimental autoimmune neuritis.

**Methods:**

Experimental autoimmune neuritis was induced in Lewis rats with the neurogenic P2-peptide. At the beginning of the recovery phase at day 18, the animals were treated with 1 mg/kg monastrol or sham and observed until day 30 post-immunisation. Electrophysiological and histological analysis for markers of inflammation and remyelination of the sciatic nerve were performed. Neuromuscular junctions of the tibialis anterior muscles were analysed for reinnervation. We further treated human induced pluripotent stem cells-derived secondary motor neurons with monastrol in different concentrations and performed a neurite outgrowth assay.

**Results:**

Treatment with monastrol enhanced functional and histological recovery in experimental autoimmune neuritis. Motor nerve conduction velocity at day 30 in the treated animals was comparable to pre-neuritis values. Monastrol-treated animals showed partially reinnervated or intact neuromuscular junctions. A significant and dose-dependent accelerated neurite outgrowth was observed after kinesin-5 inhibition as a possible mode of action.

**Conclusion:**

Pharmacological kinesin-5 inhibition improves the functional outcome in experimental autoimmune neuritis through accelerated motor neurite outgrowth and histological recovery. This approach could be of interest to improve the outcome of autoimmune neuropathy patients.

**Supplementary Information:**

The online version contains supplementary material available at 10.1186/s12974-023-02822-w.

## Introduction

Peripheral neuropathies are among the most common reason for patient referral to a neurologist [1]. Eight to ten percent of all neuropathies are of autoimmune origin, amenable to immunomodulatory therapy which in most cases halts the worsening and offers stabilisation of neuropathic symptoms. Nevertheless, even in responsive patients, a significant percentage of the patients remains with persistent disabilities and long-term need for services of the healthcare systems [2, 3]. Immune-mediated neuropathies like the Guillain–Barré syndrome (GBS) and its chronic counterpart, chronic inflammatory demyelinating polyradiculoneuropathy (CIDP), exhibit a heterogeneous presentation of axonal damage and demyelination, depending on the severity of the disease and the subtype [4]. These pathological hallmarks are also observed in experimental autoimmune neuritis (EAN), an animal model of immune-mediated neuropathies [5]. The degree of axonal damage correlates with the functional outcome as shown by electrophysiological studies or the elevated serum levels of the biomarker neurofilament light chain, which is released following axonal damage [6, 7].

The peripheral nervous system (PNS) offers a tremendous neuro-regenerative potential compared to the central nervous system (CNS), which could be exploited to ameliorate the disease burden in these patients significantly. As neurons are post-mitotic cells, neurite and axon outgrowth are the only remaining cellular correlate of neuronal regeneration. However, it is important to distinguish different types of regeneration and axonal sprouting. Sprouting derived from interrupted fibres is more complicated to achieve than “collateral sprouting” of uninjured nerve fibres [8]. For instance, focal crush injury results in a better outcome compared to nerve transection and reconnection, as it leaves the basal lamina intact and increases the probability of the sprouts to follow the path of the original fibres [8]. This leads to correct reinnervation of muscle antagonists and agonists, thus resulting in a favourable functional outcome. Acceleration and success of sprouting highly depend on motor proteins, which move along microtubules, like dynein and kinesin, supplying the nerve with energy-delivering cell organelles like mitochondria over great distances [9]. Specific modulation of these motor proteins, especially the kinesin super family, has shown remarkable in vivo and in vitro potential to promote neuronal regeneration and outgrowth [10, 11]. However, the interplay of the motor proteins and the microtubules is complex, and unselective binding can result in neuro-degeneration as displayed by the microtubule-stabilising agent paclitaxel [12]. Kinesin-5, also known as Eg5, is one of the most intriguing proteins, as its inhibition significantly accelerates both neuronal outgrowth and regeneration with an impact on the functional outcome in different in vivo studies, impacting both the CNS and PNS of juvenile and adult neurons [11, 13–17]. Kinesin-5 acts as a very slow motor, limiting the rate of any other movement along the microtubules [11]. Furthermore, the small molecule inhibitor monastrol as a cell-permeable substance allows reliably and selectively drug-induced inhibition by allosterically inhibiting the microtubule-stimulated ATPase activity of Eg5 [18, 19].

In our study, we sought to expand kinesin-5 inhibition on nerve regeneration in a monophasic model of autoimmune neuritis, where nerves are heterogeneously damaged throughout their anatomical paths, but mostly display an intact basal membrane of the original fibres. We therefore treated EAN rats with monastrol, a kinesin-5 inhibitor, after the peak of the neuritis course to improve functional outcome.

## Methods

### In vivo experiments

#### Induction of EAN

A total of 24 female, 6- to 10-week-old Lewis rats (160–200 g) were used (purchased from Charles River Co., Sulzfeld, Germany). The rats were kept under standardised conditions in our local animal facility (Medical Faculty, University Clinic of Cologne) in pathogen-free cages with food and water ad libitum. The animals were randomly divided into 2 groups with 4 animals each on the day of EAN induction, and the experiment was repeated thrice. Two animals in each group died during the anaesthetic procedures and were excluded from further analysis. The caretakers were blinded for treatment groups. Every rat was treated with tramadol (Gruenenthal, Aachen, Germany) 0.5 mg/ml per os, starting on the immunisation day, to relieve disease burden of painful autoimmune neuritis. For disease induction, the rats were anaesthetised intraperitoneally (i.p.) with xylazine (Inresa, Freiburg, Germany) and ketamine (Bayer, Leverkusen, Germany) (10 mg/kg and 50 mg/kg, respectively) and were immunised by subcutaneous injection of 250 µg of the neuritogenic P2^53−78^ peptide (#AS-65472, Anaspec, California, USA) dissolved in the same concentration of complete Freund's adjuvant (Sigma-Aldrich, Missouri, USA) into the root of the tail. Animals were weighed and assessed for disease severity daily by two independent, blinded investigators. The following clinical score was used: 0 normal; 1 less lively; 2 impaired righting/limb tail; 3 absent righting; 4 atactic gait, abnormal position; 5 mild paraparesis; 6 moderate paraparesis; 7 severe paraplegia; 8 tetraparesis; 9 moribund; 10 death [20, 21]. Termination criteria was a score ≥ 7 according to animal welfare guidelines.

#### Treatment with the kinesin-5 inhibitor monastrol

The animals were treated i.p. with 1 mg/kg monastrol, purchased from Abcam (Cambridge, UK, #ab141087, CAS Number: 329689-23-8), dissolved in 1 × phosphate-buffered saline (PBS) at day 18 post-immunisation (p.i.), day 22 and day 26. The equal volume of 1 × PBS was injected into the control group. The dosage of monastrol was derived from previous experiments [17].

#### Electrophysiological analysis

Nerve conduction studies (NCS) were performed before the immunisation at day 0, day 18 (maximum of the disease course) and day 30 (at the end of the recovery period), before the animals were killed. We followed the checklist for NCS as published [22] (Additional file 1: Material S1).

#### Immunohistochemistry

After transcardial perfusion with 1 × PBS of 6 animals per group on day 30 p.i., the right sciatic nerves were dissected into 4 segments from proximal to the distal part of the sciatic nerve and the 4 segments were immediately embedded in Tissue-Tek OCT Compound (Sakura, Tokyo, Japan) and stored in a − 80 °C freezer. The nerves were cut into 12-µm-thick slices on a cryostat (Leica Biosystems, Wetzlar, Germany) and 8 slices each were mounted on slides. We used the omission of the primary antibodies as a negative control. The specificity of the staining was also controlled on sections of peripheral lymphoid organs.

For immunohistochemical staining, cryostat sections were thawed for 2 h at 20 °C and then fixated in acetone for 20 min at 20 °C. They were washed with 1 × PBS for five minutes and then exposed to the following monoclonal antibodies (mAb) anti-Iba1 (1:500, rabbit polyclonal, FUJIFILM Wako Shibayagi Cat# 019-19741, RRID:AB_839504, Osaka, Japan) and anti-CD3 (1:500, rabbit-mAb, Abcam Cat# ab16669, RRID:AB_443425, Cambridge, UK). Corresponding fluorescein-labelled goat anti-rabbit immunoglobulin G (IgG) (Alexa Flour 488, Thermo Fisher Scientific Cat# A32731, RRID:AB_2633280, Massachusetts, USA) and anti-goat rabbit IgG (Alexa Flour 488, Jackson ImmunoResearch Labs Cat# 305-545-003, RRID:AB_2339532, Cambridge, UK) were used. We used Hoechst 33342 (1:500, Cell Signaling Technology Cat# 4082, RRID:AB_10626776, Massachusetts, USA) for cell nuclei staining. Fluorescent signals were detected using an inverted fluorescence BZ-9000 microscope (Keyence, Japan) with a 20× magnification numerical aperture objective lens (Nikon, Japan). Five pictures of each slide were randomly chosen. Cell counting per section was determined using image analysis software (ImageJ, Fiji, open source program) with a semi-quantitative approach [23].

To assess demyelination, the absence of FluoroMyelinTM Red fluorescent stain (1:300, Thermo Fisher Scientific Cat# F34652, RRID:AB_2572213, Massachusetts, USA) was assessed as published before [24]. All slides were mounted using Fluoromount G mounting-medium (Biozol, Eching, Germany).

#### Transmission electron microscopy

Animals (*n* = 4 per group) were anesthetised at day 30 via i.p. injection with 200 mg/kg body weight ketamine (Inresa, Freiburg, Germany) and 20 mg/kg body weight xylazine (Rompun, Aachen, Germany). Rats were pre-rinsed for 60 s with Tyrode’s solution, followed by perfusion fixation for 20 min with 3% glutaraldehyde + 1 mM CaCl2 in 0.1 M cacodylate buffer pH 7.4. After dissection, the sciatic nerves were sorted from proximal to distal, then postfixed in 3% glutaraldehyde, osmicated (1% OsO4), dehydrated via acetone/propylene oxide and embedded in epoxide resin (Epon, Fluka, Switzerland). Ultrathin cross sections were cut with a diamond knife (30 nm), mounted on formvar/carbon-coated 200-mesh copper grids and contrasted with uranyl acetate and lead citrate. Transmission electron microscopy was performed using a Zeiss EM109 (80 kV, 200 μm condenser and 30 μm objective apertures, 2 k TRS camera). Additional calibration of micrographs was performed through a cross-gating replica (2160 lines/mm; Polaron, England). Random images (15–20% of total nerve area) of sciatic nerves were acquired from both proximal and distal nerve sections (*n* = 4 nerves per group). The following characteristics were used: total area of the nerves in μm^2^, the estimated number of myelinated fibres per 100,000 μm^2^, fibre diameter, myelin thickness, axon diameter, and “g” ratio (“g” ratio = axon diameter/ fibre diameter) [25]. Only myelin regions that exhibited no fixation artefacts were used. The measurements were performed blinded to treatment interventions and were measured manually with ImageJ (Fiji, open source program) [26].

#### Neuromuscular junction (NMJ) staining

After transcardial perfusion with 4% paraformaldehyde (PFA), the right tibialis anterior muscles were extracted (*n* = 4 of both groups), fixed in 4% PFA over night before being cryoprotected in 30% sucrose solution over night at 4 °C. Muscles were embedded in OCT compound. Longitudinal sections of 30 µm were taken and 6 slices were mounted on a slide. After washing the slides with 1 × PBS, the tissue was permeabilised with 2% Triton X-100 for 30 min. The sections were blocked with blocking buffer (3% goat-serum, 1% Triton X-100 in 1 × PBS) for 30 min at room temperature and then incubated with primary anti-β-III tubulin antibody (1:300, rabbit-mAb, Abcam Cat# ab68193, RRID:AB_2893226, Cambridge, UK) and synaptophysin antibody (1:200, mouse-mAb, Cell Signaling Technology Cat# 9020, RRID:AB_2631095, Massachusetts, USA) over night at 4 °C. After incubation, the sections were washed with 1 × PBS three times and incubated with a mixture of α-bungarotoxin Alexa 488 conjugate (1:500, #B13422, Thermo Fisher Scientific, Massachusetts, USA) and corresponding goat anti-rabbit (1:500, Alexa Flour 568, Thermo Fisher Scientific Cat# A-11011, RRID:AB_143157, Massachusetts, USA) and donkey anti-mouse secondary antibody (1:500, Alexa Flour 568, Thermo Fisher Scientific Cat# A-11057, RRID:AB_2534104, Massachusetts, USA). Slides were then washed three times for 10 min each, and coverslips were mounted with a drop of Fluoromount G. Z-stack images were acquired using a fluorescence microscope (BZ9000, Keyence). Per animal, at least 20 NMJs were analysed for the integrity of the post- and pre-synaptic structure. NMJs with closely intact pre- and post-synaptic staining were classified as “innervated”, synapses with some endplate regions devoid of neuronal input were classified as “partially innervated”, NMJs with no pre-synaptic staining, but intact post-synapses due to axonal loss were counted as “non-innervated, intact AChR”, and no pre-synaptic structure and deformed post-synapses as “denervated” [27].

#### Blood-smear with WBC/RBC ratio

On day 30 and before transcardial perfusion, blood samples were extracted, and a blood-smear was performed. For the analysis of the white blood cell (WBC)/red blood cell (RBC) ratio, May–Grünwald–Giemsa (Sigma-Aldrich, Missouri, USA) staining was performed following the manufacturer’s protocol. Bright-field microscopy in 20× magnification (BZ9000, Keyence, Osaka, Japan) was employed. 12 × 8 mm^2^ grids were used for manual cell counting of WBCs and RBCs.

### In vitro experiments

#### Generation of human induced pluripotent stem cells (hiPSCs)-derived secondary motor neurons

HiPSCs, maintained in mTESR1 basal medium (Stemcell Technologies, Vancouver, Canada), were grown until they reached 95–100% confluency to start motor neuron differentiation. The generation of hiPSC-derived secondary motor neurons was based on a previously described protocol [28]. For differentiation, media was changed to motor neuron differentiation medium (containing DMEM/F-12/Neurobasal Medium (1:1), 1 × B27, 1 × N2-supplement, 1 × non-essential amino acids (NEAA), 1 × GlutaMAX and 1 × penicillin/streptomycin (all Thermo Fischer Scientific, Massachusetts, USA)). At day 0 (start of differentiation) until day 6, 1 µM InSolution Smoothened Agonist (SAG), 1 µM Retinoic Acid (RA) (both Sigma-Aldrich, Missouri, USA), 10 µM StemMACS SB431542 and 100 nM StemMACS LDN-193189 (both Miltenyi Biotec, Bergisch-Gladbach, Germany) were added freshly to the medium. From day 6 until day 12, the medium was supplemented with 1 µM SAG, 1 µM RA, 4 µM SU-5402 and 5 µM DAPT (all Sigma-Aldrich, Missouri, USA). On day 12, cells were split to poly-L-ornithine (PORN) (Sigma-Aldrich, Missouri, USA)/laminin (Thermo Fisher Scientific, Missouri, USA)-coated 6-well plates and differentiation medium was changed to motor neuron maintenance medium (containing Neurobasal Medium, 1 × B27, 1 × N2, 1 × NEAA, 1 × GlutaMAX, 1 × penicillin/streptomycin, 10 nM brain-derived neurotrophic factor and 10 nM glia cell line-derived neurotrophic factor (both Biozol, Eching, Germany)). Neurons were grown for seven days until further processing.

#### Treatment with the kinesin-5 inhibitor monastrol

HiPSC-derived secondary motor neurons were seeded sparsely on coverslips in a 24-well plate. Coverslips were treated for 24 h with PORN (Sigma-Aldrich, Missouri, USA) (1:80). Immediately before coating, laminin (Thermo Fischer Scientific, Missouri, USA) (1:1000) was added. Cells were then seeded with a culture medium for 48 h. Neurons were stimulated with either monastrol in concentrations of 100 µmol and 10 µmol (Cambridge, UK, #ab141087, CAS Number: 329689-23-8) dissolved in 1 × PBS; or 10 µmol 1 × PBS for another 48 h. Media change served as the control group. Cells were then fixated with 4% PFA.

#### Immunocytochemistry

For morphological analysis, fluorescent staining of the neurites was performed using 1:500 anti-MAP2-staining as a primary antibody (mouse-mAB, Sigma-Aldrich Cat# M9942, RRID:AB_477256, Missouri, USA). The wells were incubated over night at 4 °C. After washing with 1 × PBS, the cells were incubated with a donkey anti-mouse secondary antibody (1:500, Alexa Flour 568, Thermo Fisher Scientific Cat# A-11057, RRID:AB_2534104, Massachusetts, USA) for 1.5 h. For counterstaining, Hoechst 1:500 was used. After washing with 1 × PBS three times, the coverslips were mounted on slides using Fluoromount G.

#### Morphological analysis

Motor neurons were randomly selected using a fluorescence-detecting microscope (BZ9000, Keyence). Depending on the overall neuron length, either a 4×, 10× or 20× magnification was used. Only single neurons without contact with other neurons were considered for further analysis. Ten neurons of three coverslips per group were analysed, and the experiment was repeated twice. ImageJ software (Fiji, open source program) and the plugin NeuronJ were used according to the developers’ instructions [29]. A primary neurite was defined as originating directly from the soma, a secondary neurite was defined as branching from a primary neurite and a tertiary neurite as branching from a secondary neurite.

#### Statistical methods

Statistical analyses were performed by Prism software (GraphPad Prism 9, San Diego, CA). Unless stated otherwise, data are provided as mean ± SD (standard deviation). Student’s *t*-test tested differences between pairs of groups. Differences between three or more groups were tested by one-factor analysis of variance (ANOVA). In all experiments, a *p*-value of < 0.05 was defined as statistically significant, and *p* < 0.0001 was considered highly statistically significant.

## Results

### Kinesin-5 inhibition enhances the functional outcome in EAN

**Fig. 1:**
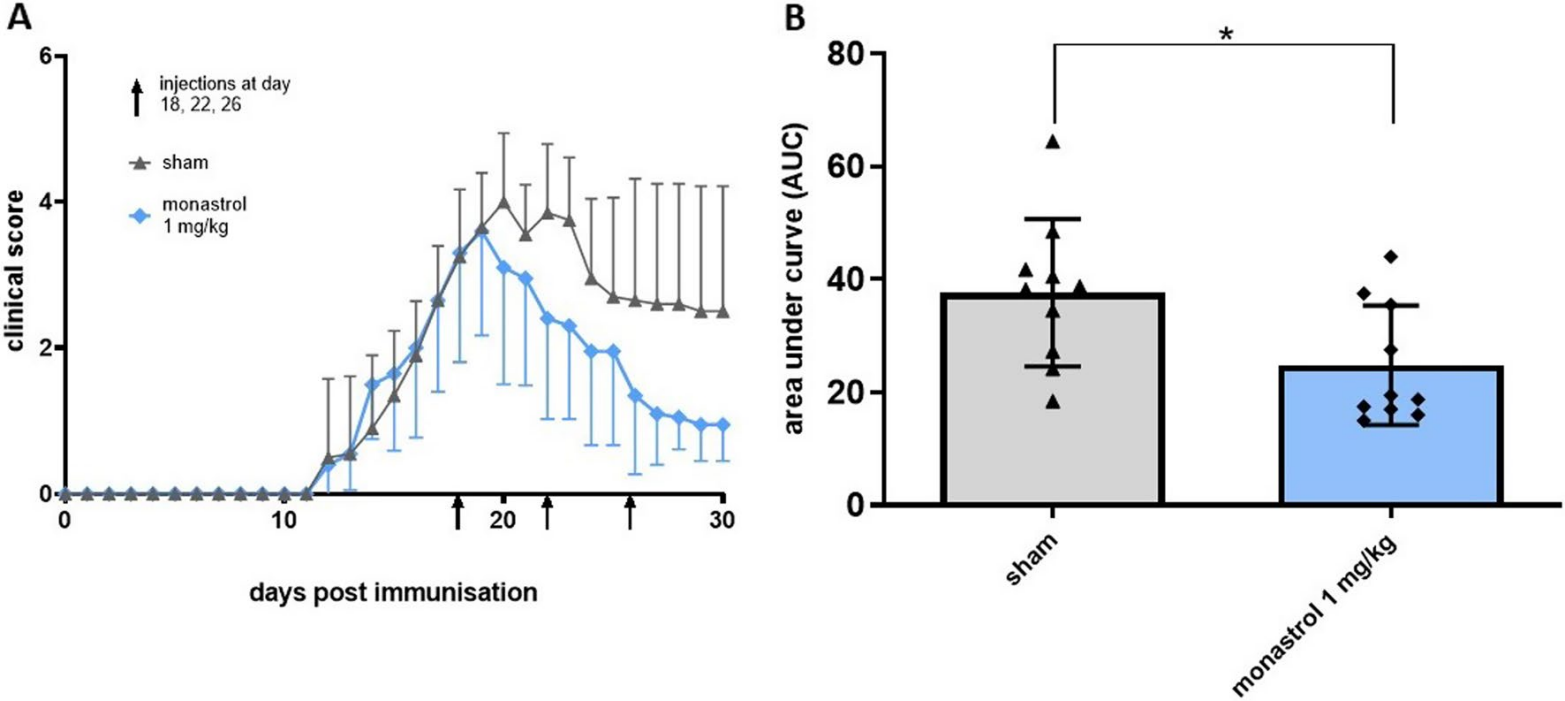
Treatment with 1 mg/kg monastrol enhances functional recovery after experimental autoimmune neuritis (EAN). EAN score from day 0 to day 30 p.i. is depicted in **A**. Overall, *n* = 10 animals were treated in each group with either 1 × PBS as control or 1 mg/kg monastrol i.p. injections at days 18, 22 and 26. Monastrol improves the EAN recovery course. Kinesin-5 inhibition reduces the area under the curve (AUC), shown in **B**. One-sample t-test was performed to row stats of data analysis, with Mann–Whitney test of AUC values. The experiment was performed three times. The SD is depicted in every picture

EAN clinical signs were seen as early as day 11 and the clinical course reached its maximum around day 19. Treatment with either monastrol 1 mg/kg or 1 × PBS was initiated with the beginning of the recovery phase at day 18, followed by repeated treatments on days 22 and 26. The incidence of EAN was 100% in the animals. The mean clinical score was 3.25 (SD ± 0.92) in the control and 3.3 in the monastrol group (SD ± 1.49) on day 19 and, therefore, comparable. The mean clinical score on day 30 was 2.5 (SD ± 1.72) and 0.95 (SD 0.5), respectively (Fig. 1A). A significant improvement of the motor symptoms was seen in the monastrol-treated rats, as shown by the area under the curve analysis from day 18 to day 30 (mean values: control = 37.68 ± 13.04, 1 mg/kg monastrol = 24.83 ± 10.56; *p-*value = 0.03) (Fig. 1B).

### Inhibition of kinesin-5 enhances motor nerve conduction velocity (mNCV) recovery

**Fig. 2:**
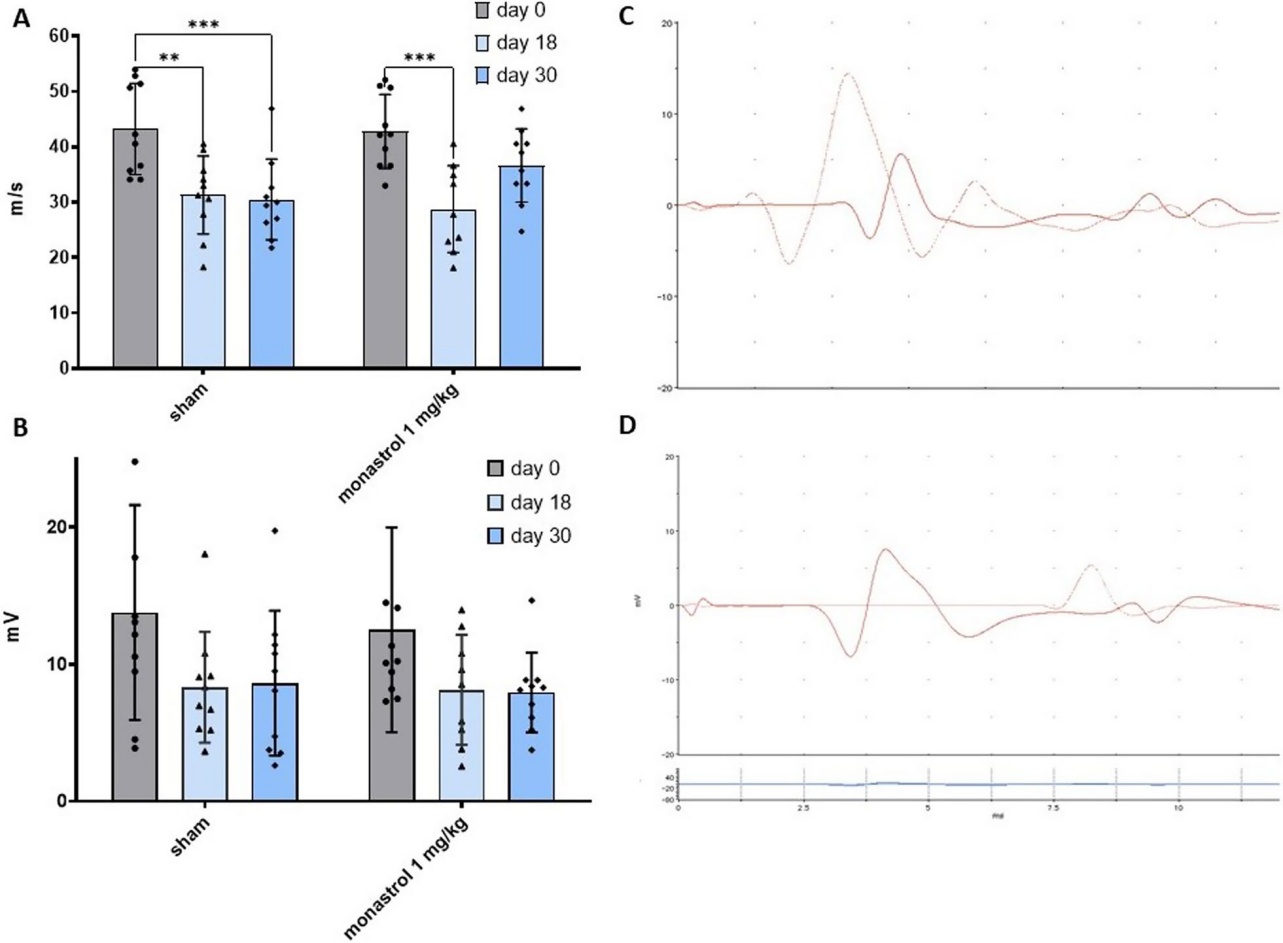
Monastrol accelerates motor nerve conduction velocity (mNCV) recovery. **A** Depicts the impact of experimental autoimmune neuritis (EAN) on mNCV at day 0, 18 and 30. The measurements of the compound action muscle potential (cMAP) of the dorsal foot are displayed in **B**. Both groups showed no significant reduction of cMAP (control group: *p*-value of comparing days 0 and 18: 0.08, days 0 and 30: 0.11; respective p-values of the monastrol group: 0.21 and 0.17; multiple 2-way ANOVA with Tukey’s multiple comparisons). **C** and **D** Show exemplary measurements of mNCV after proximal and distal stimulation at day 0 in a healthy rat in **C** and at day 18 with a prolonged mNCV in **D**. Multiple 2-way ANOVA test with Tukey’s multiple comparisons was performed. Overall, *n* = 10 rats were used. The experiment was repeated three times

Demyelination is a pathological hallmark of autoimmune neuritis, resulting in motor deficits. We further analysed whether monastrol treatment has any impact on the electrophysiologically measurable nerve function of the sciatic nerve. We conducted the mNCV and the compound muscle action potential (cMAP) as parameters for demyelination and axonal damage at days 0, 18 and 30 p.i. of the sciatic nerve. Mean mNCV at day 0 was comparable in both groups (control: 43.21 m/s ± 8.2 SD; monastrol 42.78 m/s ± 6.68 SD), with a significant reduction of mNCV at day 18 compared to day 0 (*p*-value: 0.002) in the control group (31.3 m/s ± 7.04 SD) and the monastrol group (28.76 m/s ± 7.85 SD, *p*-value: 0.0003 compared to baseline). Treatment with monastrol accelerated recovery of mNCV, with a mean 36.64 m/s (± 6.64 SD) at day 30 to near baseline value (*p*-value: 0.1819), while no changes could be observed in the sham group (30.50 m/s ± 7.3 SD, *p*-value: 0.0008 compared to baseline, Fig. 2A).

CMAP measurement of the dorsal foot muscles showed a non-significant reduction in both groups from day 0 to day 18 (control: at day 0 13.79 mV ± 7.86 SD, at day 18 8.34 mV ± 4.05 SD, and for monastrol: at day 0 12.53 mV ± 7.48 SD, at day 18 8.13 mV ± 4.01 SD) and no improvement in both groups at day 30 (control: 8.64 mV ± 5.29 SD and for monastrol: 7.94 mV ± 2.91 SD, Fig. 2B).

### Monastrol does not significantly alter myeloproliferation

**Fig. 3:**
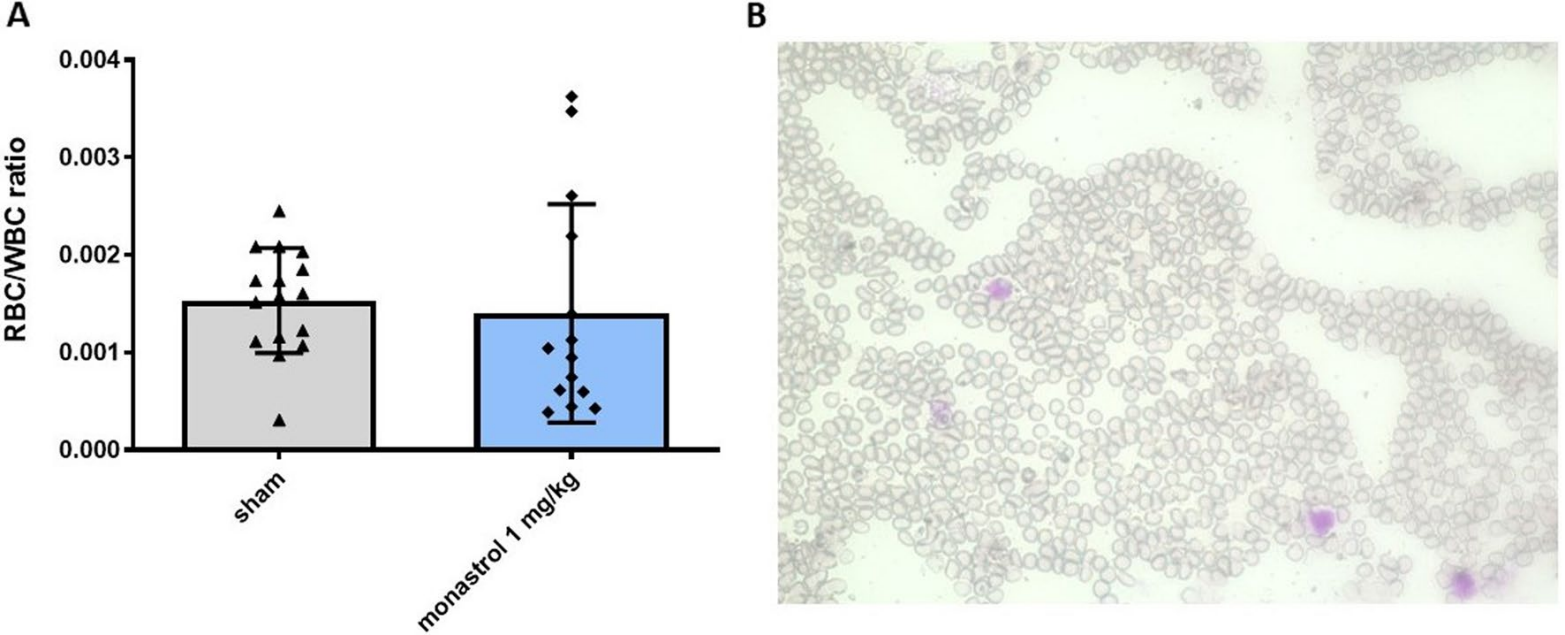
Red blood cell and white blood cell count ratio (RBC/WBC) count at day 30. **A** Shows the RBC/WBC ratio of the control and the monastrol 1 mg/kg group (*n* = 4 animals per group). **B** Shows a standard May–Grünwald–Giemsa staining of a rat treated with monastrol. The red blood cells without a nucleus can be easily identified. Larger white blood cells are marked purple. An unpaired two-tailed t-test was performed. The standard deviation is depicted in **A**

A possible side-effect of inhibiting microtubule-interacting motor protein kinesin-5 is its myelosuppressive potential. This could significantly impact fast-dividing cells, e.g., blood cells. For further analysis, we performed a blood-smear at day 30 of *n* = 4 rats in each group and calculated the white blood cell and red blood cell ratio. There was no difference between the two groups (mean ratio for control: 0.0015 ± 0.0005 SD; mean ratio for monastrol: 0.0014 ± 0.011 SD; *p*-value 0.68, Fig. 3).

### Kinesin-5 inhibition significantly enhances remyelination and reduces T-cell infiltration

**Fig. 4:**
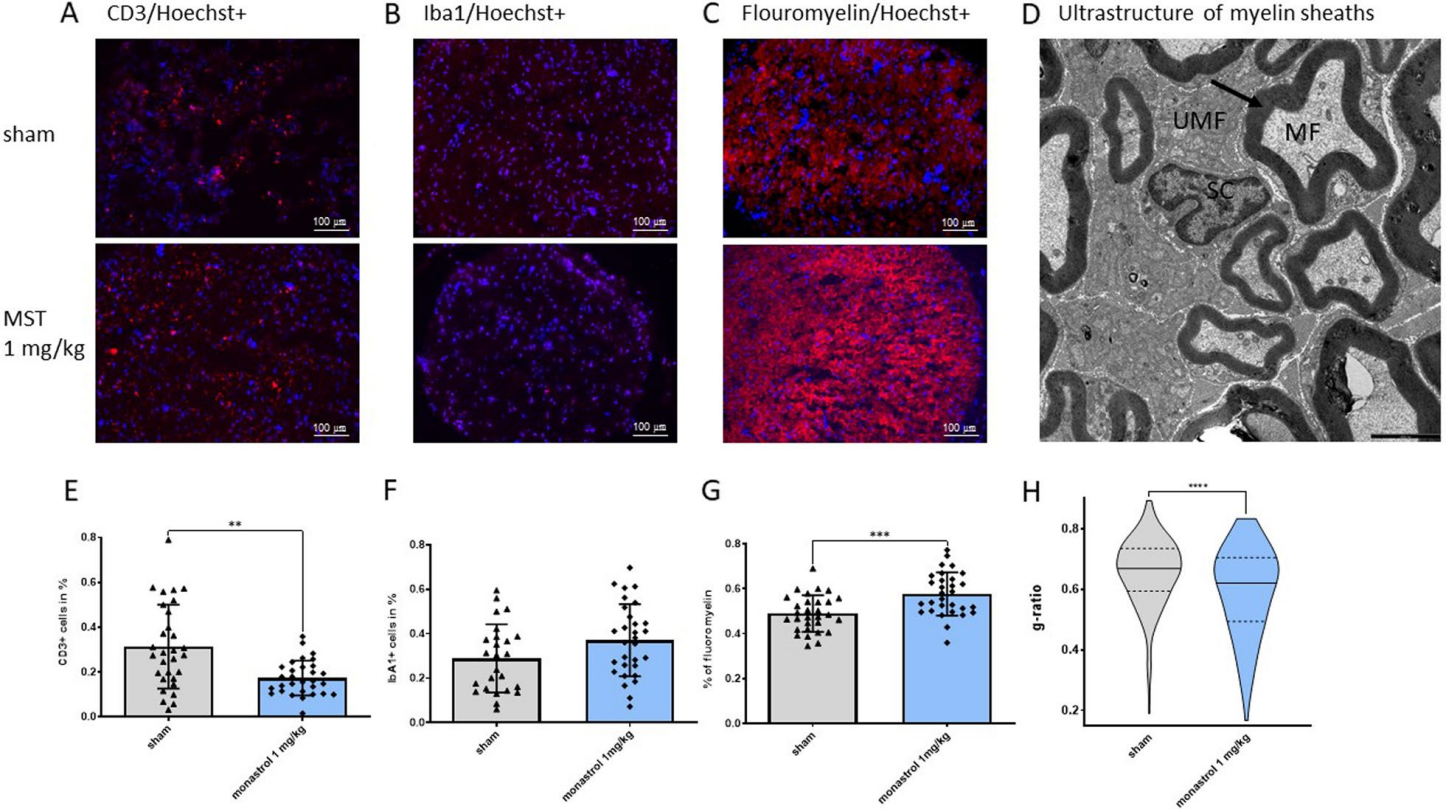
Immunostaining for the cellular inflammation markers CD3^+^/Iba1^+^, semi-quantitative myelin-staining and ultrastructural analysis of myelinated fibres. **A** and **E** depict the infiltration of CD3^+^ cells and **B** and **F** for Iba1^+^ cells in the sciatic nerves of the control and monastrol (MST) group. Fluoromyelin staining is shown in **C** and **G**. For each performed immunostaining, we analysed *n* = 25–30 of 10 nerves per group. In** D**, transmission electron microscopy of the sciatic nerves is shown. Exemplary, a myelinated fibre was marked with MF; unmyelinated fibres with UMF. In the middle of the image, a Schwann cell is depicted (SC). The black arrow indicates the myelin sheath; the scale is shown in black (2500 nm). Overall, 7–10 images of each nerve’s proximal and distal parts were randomly taken in each group (*n* = 4 nerves per group, resulting in 264 myelin sheaths for control and 186 fibres for MST). Myelinated fibres of MST-treated rats had a significantly lower g-ratio (axon diameter/ fibre diameter) (**H**, depicted as a truncated violin plot with the median and quartiles). For statistical analysis, an unpaired two-tailed t-test was performed. The standard deviation is depicted in **E**, **F** and **G**

To address histological severity and immunological recovery at day 30 of EAN, we quantified markers of cellular inflammation of the sciatic nerve via immunohistochemistry. CD3^+^ cells were significantly reduced in monastrol-treated animals (mean ratio for control: 0.31% ± 0.19 SD, for monastrol: 0.14% ± 0.08 SD, *p*-value: 0.001) while anti-IbA1-staining showed more infiltration in the treatment group, albeit not statistically significant (control: 0.29% ± 0.154, monastrol: 0.37% ± 0.16, *p*-value: 0.06). For histological analysis of remyelination, semi-quantitative fluoromyelin-staining was highly significant in favour of the monastrol 1 mg/kg group (control: 0.49% ± 0.08, monastrol: 0.58% ± 0.1, *p*-value: 0.0005). We further accessed remyelination on an ultrastructural level. No significant differences in the mean fibre diameter between the groups were found (mean fibre diameter for control: 4494 nm ± 2218 nm, for monastrol: 4301 nm ± 2420 nm, p-value: 0.16). The axon diameter was reduced in the control group (mean 2976 nm ± 1587 nm to 2629 ± 1718 nm, *p*-value 0.0031), with an, albeit not significant, lower total nerve area of a mean 19.93 ± 37.49 μm^2^ (monastrol: 24.35 ± 68.8 μm^2^). The group treated with 1 mg/kg monastrol had significant thicker myelin sheaths of myelinated fibres in the sciatic nerve (mean thickness of myelin sheath in control: 647.6 nm, in monastrol: 937.1 nm, *p*-value: 0.0002), and a concordant highly significant decrease of the g-ratio (mean ratio for control: 0.66 ± 0.11, for monastrol 1 mg/kg: 0.59 ± 0.15, *p*-value < 0.0001) as an indirect sign of remyelination (Fig. 4). The estimated number of myelinated fibres per 100,000 μm^2^ was comparable (control: 1907 ± 562, monastrol: 1561 ± 745, *p*-value: 0.08).

### Kinesin-5 inhibition accelerates motor neuron outgrowth

**Fig. 5:**
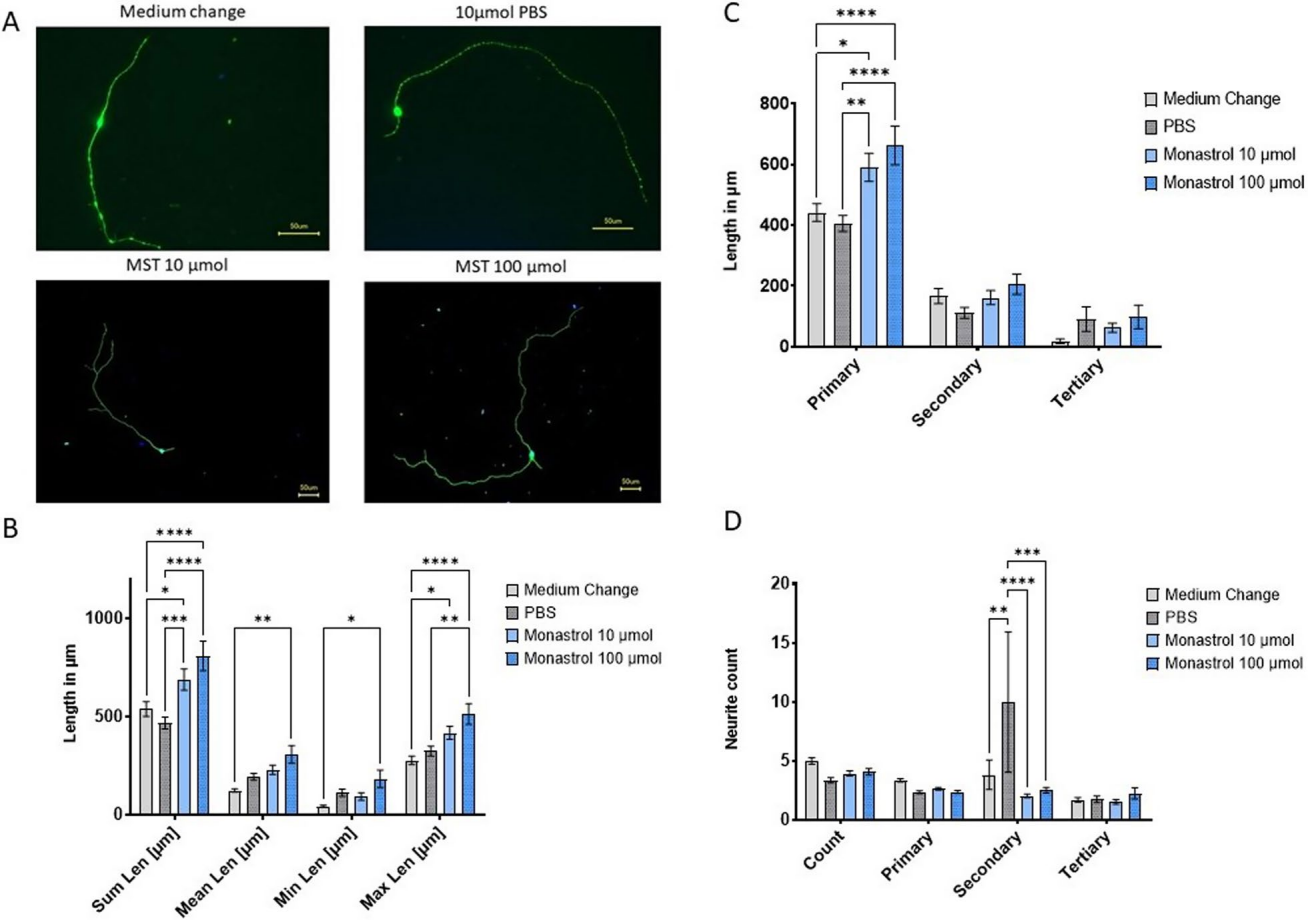
Assessment of the outgrowth of human induced pluripotent stem cells-derived secondary motor neurons. Exemplary immune-histochemical staining of the treatment groups is shown in **A**. MAP2^+^ staining allows morphological analysis of neurites, and Hoechst was used for cell body staining. In **B**, the neurite’s overall, mean, minimal and maximal length are shown. Overall neurites length significantly increased compared to controls (medium change: 537.16 ± 37.17 SEM; PBS: 466.47 ± 29.75 SEM) with monastrol (MST) treatment (monastrol 10 µmol: 687.36 ± 53.87 SEM; monastrol 100 µmol: 808.34 ± 75.22 SEM), mainly due to a length increase of the primary neurites as depicted in **C** (medium change: 441.63 ± 29.18 SEM; PBS: 405.54 ± 26.22 SEM; monastrol 10 µmol: 590.36 ± 46.33 SEM; monastrol 100 µmol: 662.84 ± 63.37 SEM).** D** Shows the overall neurite count (mean count for medium change: 4.96 ± 0.28 SEM; PBS: 3.33 ± 0.22; monastrol 10 µmol 3.9 ± 0.22 SEM and monastrol 100 µmol 4.05 ± 0.29). Overall, between 100 and 103 neurons were analysed per group. For statistical analysis, multiple 2-way ANOVA with Tukey’s multiple comparisons, was used. SEM is displayed in **B**–**D**

Kinesin-5 inhibition accelerates the neuronal growth of primary sensory neurons and primary motor neurons in rats and mice [11, 13–15]. However, to our knowledge, there is no study examining the impact of monastrol treatment on secondary motor neurons, the neuron type mainly affected in autoimmune neuritis and of interest for functional outcome and recovery. We, therefore, treated hiPSCs-derived secondary motor neurons with monastrol in different concentrations for 48 h and performed a complexity analysis via neurite staining. Both concentrations of monastrol led to a highly significant and dose-dependent increase of the overall neurite length (Fig. 5B) (*p*-values of monastrol 10 µmol vs PBS: 0.0001, vs medium change: 0.02 and *p*-values of monastrol 100 µmol vs PBS and vs medium change, respectively: < 0.0001). The effect was mainly attributed to an increase of the longest neurite and the primary neurites (Fig. 5C) (*p*-values of monastrol 10 µmol vs PBS: 0.002, vs medium change: 0.02 and p-values of monastrol 100 µmol vs PBS and vs medium change, respectively: < 0.0001) and not to an increase in branching (Fig. 5D).

### Monastrol enhances reinnervation of the NMJs during the recovery phase of EAN

There is growing evidence that the pre-synaptic membrane of the NMJ and the peri-synaptic Schwann cells are targeted by anti-ganglioside antibodies in GBS [30]. Degeneration is mediated by complement activation [31, 32], which is also induced in EAN [33, 34]. However, instead of anti-ganglioside antibodies, EAN is induced by immunisation against myelin-antibodies, in this case, P2-peptide. Even though terminal axons lose myelin in EAN, it is unclear if the NMJ is also targeted [35]. Our in vitro experiment reveals that kinesin-5 inhibition accelerates secondary motor neurite outgrowth, suggesting a pivotal impact on muscular reinnervation. We, therefore, decided to analyse further the post- and pre-synaptic integrity of the tibialis anterior muscle, innervated by the sciatic nerve.

**Fig. 6:**
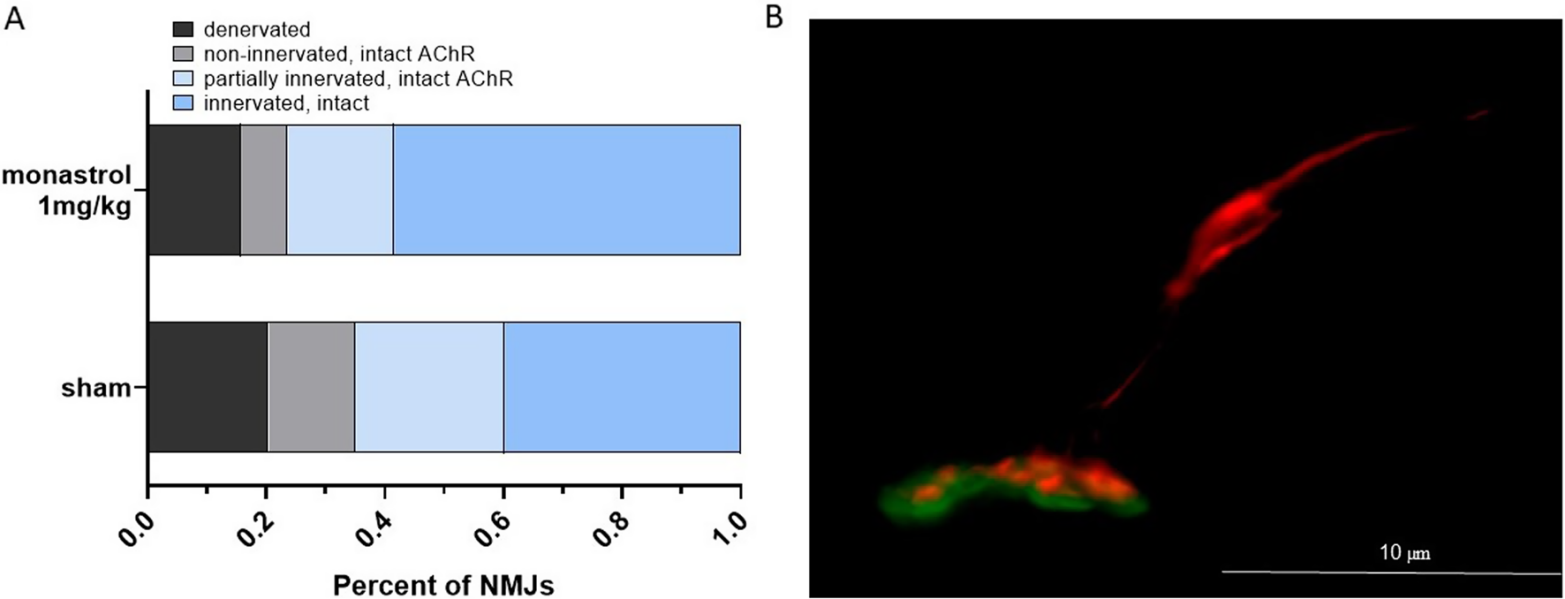
Innervation status of the neuromuscular junctions (NMJs) at the end of the recovery phase. Immunohistochemical analysis via Tuj1 and synaptophysin displayed the pre-synaptic formation, while the post-synapsis was α-bungarotoxin positive. The shift to (partially) reinnervated NMJs is shown in **A**. In **B**, an innervated NMJ of the monastrol group is depicted. The post- and pre-synaptic structures are nearly overlapping. Overall, 89–103 NMJs were analysed per group (*n* = 4 animals per group)

At day 30, we observed in both groups denervated or partially denervated NMJs, providing evidence that EAN impacts the NMJ. Monastrol-treatment led to more intact or partially innervated NMJs (control: 65.05% versus monastrol: 76.4%, Fig. 6).

## Discussion

GBS, as the most common acute autoimmune neuropathy induces long-term deficits in around 20% of the patients, with muscle atrophy from denervation and only incomplete nerve regeneration and reinnervation [36]. Besides, patients suffer from both positive sensory symptoms like pain and negative sensory deficits. Over 1/3 of all GBS patients report severe pain one year after disease onset, which can persist over 10 years [37–39]. First-line immunomodulatory therapies are immunoglobulins and plasma exchange, and both treatments hasten the recovery of GBS [40]. However, effects on the long-term outcome are inconclusive, as GBS functional outcome prognosis seems to be more dependent on disease severity, patient age and disease subtype [41, 42].

Up to date, only limited preclinical studies are available, that focus on improving neuro-regeneration in GBS or its rodent models. Experimental set-ups often concentrate on diminishing the inflammatory reaction or promoting neuro-protection. In EAN, the time-point of disease induction is known and neuro-protective or anti-inflammatory treatments start during the disease induction phase or with the beginning of the first symptoms [43]. In GBS, an induction phase also occurs, as several GBS patients report an infectious event weeks before symptom onset. This is most evident in *Campylobacter jejuni* gastroenteritis-associated GBS [2]. However, only around 1 out 5000 of these gastroenteritis patients proceeds to develop a GBS [2, 40]. It is currently impossible to predict patients at risk and examine or influence the disease course during the induction phase. This shortcoming results in one of the major translational pitfalls of preclinical studies about neuro-protection in rodent models of autoimmune neuritis.

In this study, we aimed to specifically observe and influence neuro-regeneration in a pure autoimmune neuritis model, focusing solely on the recovery phase to distinguish neuro-protection from neuro-regeneration. Through the pharmacological inhibition of the motor protein kinesin-5, we could enhance the functional and histological recovery in autoimmune neuritis. We provide evidence from both in vivo and in vitro experiments, using the cell-permeable small molecule monastrol at concentrations of 1 mg/kg in vivo.

Despite having a comparable disease severity at day 18 p.i., the motor symptoms significantly ameliorated with monastrol treatment and mNCV, semi-quantitative myelin-staining, and ultrastructural analysis of myelin sheaths indicated a faster remyelination of the nerves, as well as a higher reinnervation of the NMJs at day 30 (Figs. 1, 2 and 6).

We could show that monastrol significantly and dose-dependently accelerates neural outgrowth in vitro. Notably, this effect was not limited to primary motor or sensory neurons, but extended to secondary human motor neurons. Our morphological analysis showed that the overall length increase was mainly mediated by an increase in the primary neurites and not by an overt branching of the neurites (Fig. 5). MAP2-staining does not allow for distinguishing between axons and dendrites, but as nearly all hiPSC-derived secondary motor neurons displayed only two primary neurites, the primary neurites are the axons in these neurons. We, therefore, conclude that kinesin-5 inhibition exhibits its neural outgrowth-enhancing potential mainly by affecting the axon, which was also described in another study [44].

Several studies showed that the specific inhibition, overexpression and knock-out of motor proteins have promising potential to positively impact neuronal regeneration [14, 45, 46]. However, the specificity of the targeted motor protein is highly relevant, as therapy-limiting side-effects of unspecific or incorrect motor protein- and microtubule-interacting agents like paclitaxel are their neurotoxicity, and neurons are especially dependent on intracellular movement rates [47]. Kinesin-5 has been shown to act as a brake of intracellular axonal transport and its inhibition enhanced neuronal outgrowth in vitro of primary motor and sensory neurons in both the juvenile and adult state. It remains to be elucidated whether this effect translates into a meaningful functional improvement under pathological conditions [11, 13, 14, 16, 48]. We could show that monastrol treatment prevents bortezomib-induced neurotoxicity in vivo [17]. Xu et al. [15] described the effects of monastrol in a model of a peripheral nerve graft transferred for spinal cord injury (SCI). Their data suggest that a combinatory treatment of chondroitinase ABC and monastrol significantly enhanced axonal regeneration, albeit without functional improvement.

Secondary motor neurons are involved in both Xu et al. and our model, but EAN targets the PNS, not the CNS. SCI results in degeneration of the secondary motor neuron but also involves distal parts of primary motor neurons, sensory neurons, and interneurons [49, 50]. Conversely, in EAN, Wallerian degeneration is only irregularly reported and associated with higher antigen dosages [51, 52]. Notably, with an intact basal membrane, the anatomical pathway for neurite outgrowth is still available, resulting in the correct reinnervation of muscle agonists and antagonists [8]. Incorrect reinnervation is a significant pitfall of sciatic transection models resulting in a disappointing functional outcome even with near-complete neuronal recovery [53, 54]. The severity of EAN in our animals was modest with only a few animals suffering from severe or moderate paraplegia, indicating that Wallerian degeneration did—if at all—only occur infrequently in our experimental setting. Our ultrastructural nerve fibre analysis, in which Wallerian degeneration was rarely observed, supports this assumption.

We observed a significant decrease of CD3^+^ T cells in the sciatic nerve after monastrol treatment without any effects on macrophage infiltration (Fig. 4), indicating a faster histological recovery. T cells play a critical role in the disease induction phase, as antigen-specific T cells invade the blood–nerve barrier, whereas macrophages are the crucial component for the final effector phase [55, 56]. Monastrol qualifies as a chemotherapeutic and has possible side-effects on fast-dividing cells, e.g., blood cells. As no clinical (human) studies for monastrol are available, we explored possible side-effects on myeloproliferation. Analysis of the white/red blood cell ratio showed that the concentration of 1 mg/ml used in this study did not significantly impact myeloproliferation (Fig. 3).

As we first applied kinesin-5 inhibition at the beginning of the recovery phase, starting at day 18 [57], direct anti-inflammatory effects of monastrol on immune cells involved in EAN pathogenesis appear unlikely.

Two more recent studies examined modulatory effects on neuro-regeneration in another GBS mouse model [58, 59]. In the study of Asthana et al., forced expression of human heat shock protein 27 extended the critical period for muscle reinnervation to regain functional motor recovery by preserving distal axonal ends from degeneration [58]. In contrast to our methods, Asthana et al. used a combinatory approach in which a sciatic nerve crush was followed up by anti-ganglioside antibody injections to induce GBS. Transgenic mice that constantly overexpressed human heat shock protein 27 were used in that study, making it challenging to distinguish neuroprotective from neuro-regeneration-promoting effects. The same group used the combinatory model of nerve crush and anti-ganglioside antibodies to examine erythropoietin effects [59]. In that study, erythropoietin reversed the inhibitory effects of anti-ganglioside antibodies on axon regeneration in cell culture models and significantly improved nerve regeneration in the animal model.

Regarding the positive and negative sensory deficits displayed in GBS and EAN, Wei et al. [16] found compelling evidence that kinesin-5 inhibition reversed pathological pain by inhibiting vanilloid receptor subtype 1 (VR1) axonal trafficking. Painful sensory deficits also occur during autoimmune neuritis, indicating another possible potential indication for monastrol [60]. This warrants further investigations, which should not only include a thoroughly motor behavioural testing, but also testing of the sensory function for pain, temperature and touch.

Our study displays several limitations. First, the observed severity of EAN in our experiments was moderate. In severe cases of EAN and GBS, there is almost complete axonal degeneration with destruction of the basal membrane [61]. It remains unclear, whether kinesin-5 inhibition could possibly impact functional recovery under these conditions.

Second, the safety data of motor-protein inhibition are unclear. Beneficial effects on nerve regeneration of kinesin-5 inhibition might adversely affect nervous tissues like the brain. For example, kinesin-5 inhibition may contribute to neuro-degeneration of Alzheimer’s disease and impair dendritic morphology [11, 48, 62]. Further studies are needed to exclude bone marrow suppressive side-effects, e.g., differential analysis of peripheral blood and bone marrow cells, as our study provides only explorative data.

Lastly, the effects of monastrol on glia cells like Schwann cells or satellite glia cells are unknown. Both cell types are involved in pro- and anti-regenerative events [63, 64]. MNCV recovery and immune-histochemical and ultrastructural analysis suggest accelerated remyelination in sciatic nerves due to monastrol treatment. However, little is known about kinesin-5 expression in Schwann cells and satellite glia cells. Further studies are needed to address these uncertainties.

## Conclusion

Our results show that kinesin-5 inhibition via the small molecule monastrol constitutes a promising pharmaceutical target to accelerate neuronal recovery and improve the outcome in autoimmune neuritis. Further studies are needed to address the translational value of kinesin-5 inhibition and monastrol for autoimmune neuropathy in patients.

## Supplementary Information

**Additional file 1.** Material S1.

## Data Availability

The datasets used and/or analysed during the current study are available from the corresponding author upon reasonable request.

## Abbreviations

AUC: Area under the curve
CIDP: Chronic inflammatory demyelinating polyneuropathy
CNS: Central nervous system
EAN: Experimental autoimmune neuritis
GBS: Guillain–Barré syndrome
HiPSCs: Human induced pluripotent stem cells
MNCV: Motor nerve conduction velocity
NCS: Nerve conduction studies
NEAA: Non-essential amino acids
NMJ: Neuromuscular junction
PBS: Phosphate-buffered saline
PFA: Paraformaldehyde
PNS: Peripheral nervous system
PORN: Poly-L-ornithine
RA: Retinoic acid
SAG: Smoothened agonist
SCI: Spinal cord injury
SD: Standard deviation
SEM: Standard error of the mean
