# Supplementary material for: Kinesin-5 inhibition improves neural regeneration in experimental autoimmune neuritis
Source: J Neuroinflammation. 2023 Jun 9;20:139. doi: 10.1186/s12974-023-02822-w (PMC10257330; doi:10.1186/s12974-023-02822-w)
Supplement: Supplementary file 1 — Additional file 1. Material S1. [file 12974_2023_2822_MOESM1_ESM.docx]

**Additional file Material S1**

1) Animals Species

We used female Lewis rats, 6-10 weeks old, which weighted 160-200 g at day 0.

2) Anaesthesia

The rats were anaesthetised intraperitoneally (i.p.) with xylazine and ketamine (10mg/kg and 50mg/kg, respectively).

3) Temperature control

Measures were performed on a temperature-regulated plate (37 ± 1 °C), and temperature was controlled via a rectal probe.

4) Electrode Type

We performed electrophysiological studies with a PowerLab signal acquisition set-up (ADInstruments).

5) Placement / Positioning

The right sciatic nerve was used for electrophysiological analysis. Paired needle electrodes were inserted at the sciatic notch or the popliteal fossa, and the sciatic nerve was stimulated. The resulting compound muscle action potential (cMAP) was recorded from needle electrodes placed subcutaneously over the dorsal foot muscles. A ground electrode was placed at the tail. Motor nerve conduction velocity was calculated by dividing the distance between stimulating cathodes of both proximal and distal stimulation by the difference in the latency (35 mm).

6) Pulse amplitude/duration

The used pulse amplitude was 20 mA and the stimulation duration 50 ms.

7) Data reporting

See results.
